# Retraction: Ramentaceone, a Naphthoquinone Derived from *Drosera sp*., Induces Apoptosis by Suppressing PI3K/Akt Signaling in Breast Cancer Cells

**DOI:** 10.1371/journal.pone.0226703

**Published:** 2019-12-12

**Authors:** The PLOS ONE Editors

After publication of this article [1], concerns were raised about results reported in Figs 2, 3, 5, 6, 7 and 8, as detailed below. In light of some of these, an Expression of Concern was issued by the *PLOS ONE* Editors on January 29, 2019 [2].

Since, the authors stated that a third-party company conducted the colony assay, western blot, and flow cytometry experiments reported in the article, but that initial colony assay and western blot replicates were done in the authors’ laboratory. According to the authors, the results reported in Figs 2, 3, 5, 6, 7, 8 were provided by the third-party company, and the authors do not have the underlying data supporting these figures.

The authors did not provide data from the original experiments or comments to address the specific concerns about Figs 2, 3, 5, 6, 7 and 8, but the company provided clarifications and supporting files. Specific concerns include:

- In Fig 2B, similarities were noted between the 0 and 0.5 μM panels for BT474, and between the 0.5 and 1 μM panels for SKBR3. A representative of the third-party company clarified that the BT474 0.5 μM image was mistakenly included as also representing the BT474 0 μM condition. Furthermore, an image showing the plate from the SKBR3 0.5 μM experiment after an additional wash step was errantly reported as representing the SKBR3 1 μM experiment. Original images were provided to support the Fig 2B results.
- Several concerns were raised about similarities in FACS plots:
  ○ Fig 5: Similarities were noted between several regions of the MCF-7 panels, and separately between several regions of the MDA-MB-231 panels.
  ○ Similarities were noted between regions of the BT474–10 panel of Fig 5 and the BT474 –Akt siRNA/5 μM ramentaceone panel of Fig 8B.
  ○ Similarities were noted between regions of the SKBR3–10 panel of Fig 5 and the SKBR3 –ctrl siRNA/5μM ramentaceone panel of Fig 8B.
  ○ Fig 8B: Similarities were noted between regions of three BT474 panels (ctrl siRNA non-treated, cntrl siRNA 5 μM ramentaceone, Akt siRNA non-treated), and between regions of three SKBR3 panels (cntrl siRNA non-treated, Akt siRNA non-treated, Akt siRNA 5 μM ramentaceone).

The company representative commented that raw data files do not include overlaying cells. Supporting files were provided by the company but have not undergone a full editorial review in light of the other issues raised in this case. As such, the concerns about the above FACS plots have not been verified.

- When adjusted for brightness and contrast, similarities between western blot background pixilation patterns were noted between BT474 and SKBR3, results shown in Fig 7 (Bax, Bak, Bcl-2, and β-actin panels), and between several additional western blot panels in Figs 3, 6C and 8A. The company representative clarified that in assembling the panels, the respective bands were cropped and placed onto background templates, i.e. the background image in each figure panel does not represent the background as apparent on the original data. Common templates were used and thus there is overlap in background between panels. The company representative provided some files in support of the western blot results, but these files did not resolve the concerns about how data were reported or clarify the validity of the results. The western blot concerns also have implications for the validity of the quantitative data reported in Figs 3, 6 and 8.

The contributions of the third-party company were not disclosed in the published article. Hence, the listed author contributions do not fully and accurately describe who conducted the reported experiments.

In response to these issues, the authors provided replication data in support of the results in question. Nevertheless, in light of the confirmed concerns about western blot data reporting and substantial undisclosed contributions by a third-party company to the reported work, the *PLOS ONE* Editors retract this article.

AK and EL did not agree with retraction.
